# Factors associated with severity of naturally occurring piscirickettsiosis in netpen‐ and tank‐reared juvenile Atlantic salmon at a research aquarium in western Canada

**DOI:** 10.1111/jfd.13102

**Published:** 2019-11-10

**Authors:** Simon R. M. Jones, Amy Long, Christine MacWilliams, Mark Polinski, Kyle Garver

**Affiliations:** ^1^ Fisheries and Oceans Canada Pacific Biological Station Nanaimo BC Canada

**Keywords:** atlantic salmon, mitigation, piscirickettsiosis, risk factors

## Abstract

The opportunistic examination of factors associated with an outbreak of piscirickettsiosis (SRS) is described in Atlantic salmon *Salmo salar* post‐smolts held in an open netpen or in tanks supplied with raw sea water at a research aquarium in western Canada. During the outbreak, seawater temperature was significantly higher and salinity significantly lower in the netpen compared with the tanks. Mortality in the netpen began approximately 3 weeks prior to that in the tanks, and cumulative mortality in the netpen (34%) was significantly higher than in the tanks (12%). *Piscirickettsia salmonis* was confirmed by qPCR in tissues from moribund and dead fish and from colonies grown on enriched blood agar medium. Neither *P. salmonis* nor SRS were observed in salmon held concurrently in UV‐irradiated sea water. The elevated mortality was curtailed by treatment with oxytetracycline. These observations further indicate warmer, less saline and periodically hypoxic seawater are risk factors for SRS. UV irradiation of sea water is shown to be a tool for SRS management in fish‐holding facilities.

## 1 INTRODUCTION

Piscirickettsiosis is an acute septicaemic disease of farmed marine fish caused by infection with the facultative, intracellular, Gram‐negative bacterium *Piscirickettsia salmonis* (Fryer, Lannan, Giovannoni, & Wood, 1992; Rozas & Enriquez, 2014). Salmonid piscirickettsiosis (SRS) has been reported from Chile, Norway, Scotland, Ireland, and from the east and west coasts of Canada. The severity of SRS varies among geographic localities and host species, and the disease is considered emergent and of economic significance (Rozas & Enriquez, 2014).

In western Canada, a rickettsial septicaemia was reported in pink salmon (*Oncorhynchus gorbuscha*) reared on raw sea water at a research aquarium in Nanaimo in 1970 and 1978 (Evelyn, Kent, Poppe, & Bustos, 1998). Neither the origin of the infections nor its prevalence was reported. Injection of infected tissue from the pink salmon caused mortality in pink, Chinook (*O. tshawytscha*) and coho (*O. kisutch*) salmon, and the bacterium was transmitted to naïve cohabitants from infected pink salmon (Evelyn et al., 1998). The first well‐described SRS outbreak in western Canada occurred in Atlantic salmon (*Salmo salar*) at a marine netpen aquaculture site near Vancouver Island in 1991 (Brocklebank, Evelyn, Speare, & Armstrong, 1993; Brocklebank, Speare, Armstrong, & Evelyn, 1992). The cumulative mortality during this outbreak was 8%, and *P. salmonis* was confirmed as the causative agent by both immunological and molecular methods (Mauel, Giovannoni, & Fryer, 1996, 1999). Between 2002 and 2016, there were 36 farm‐level diagnoses of SRS in western Canada (Jones, 2019).

The present study describes the opportunistic examination of factors associated with the severity and eventual resolution of a naturally occurring outbreak of SRS among captive Atlantic salmon post‐smolts held in tanks or an open netpen at a marine research facility in western Canada. In addition, we report the occurrence of the infection in wild pink salmon and suggest this species to be a reservoir of infection.

## 2 MATERIALS AND METHODS

### 2.1 Fish husbandry and study design

Vaccinated (Ermogen DIP^®^, Vibrogen 2^®^, Alphaject Micro 4^®^, Apex‐IHN^®^) Atlantic salmon smolts (150 g) were obtained from a commercial hatchery on Vancouver Island and transported in aerated freshwater to the research aquarium at the Pacific Biological Station (PBS), Nanaimo, British Columbia. The seawater supply for the PBS aquarium is pumped from approximately 2 m below the low tide datum in Departure Bay and sand‐filtered (RSW). RSW was ultraviolet‐irradiated (UVSW; mean dose: 426.2 mJ/cm^2^, range: 317–721 mJ/cm^2^; peak *λ*: 265 nm, range: 240–280 nm). The fish were smolted onto UVSW in 6500‐L flow‐through tanks and acclimated for 2 weeks during which they were fed a commercial pelleted diet at a daily rate of 1% biomass. The photoperiod reflected ambient conditions. Husbandry protocols followed guidelines of the Canadian Council on Animal Care, and the study was approved by the Pacific Region Animal Care Committee (AUP#18‐010). Fish were transferred to duplicate 4,300 L of either RSW (*n* = 300/tank) or UVSW (*n* = 145/tank), with water flows of 35–40 L/min to all tanks. A third group (*n* = 700) was maintained in an open netpen (4.6 × 4.9 × 4.6 m) secured to Brandon Island in Departure Bay, approximately 500 m from the aquarium seawater intake. Initial biomass density was 5.1 kg/m^3^ in UVSW tanks, 10.5 kg/m^3^ in RSW tanks and 1.0 kg/m^3^ in the netpen. All fish received a daily ration of pelleted feed at 1% biomass. Dissolved oxygen (DO), water temperature and salinity were measured daily along with feeding performance and mortality. Moribund and dead fish were submitted to the PBS Diagnostic Laboratory for routine necropsy.

### 2.2 Necropsy

Imprints of fresh kidney and liver were made on glass microscope slides, air‐dried, fixed in 95% ethanol and stained with Giemsa or Gram stains. Alternatively, the imprints were heat‐fixed, post‐fixed in acetone and screened for *Renibacterium salmoninarum* by using the direct immunofluorescent antibody test (DFAT). They were incubated with a bacterium‐specific FITC‐conjugated goat antibody (KPL) and examined by fluorescent microscopy, as previously described (Pascho, Elliott, & Streufert, 1991). Fresh liver and kidney were streaked onto BCG (Mauel, Ware, & Smith, 2008) plates and all plates incubated at 15°C for at least 7 day. Any bacterial growth was subcultured onto BCG and individual colonies sampled for qPCR. Replicate liver samples were preserved in 95% ethanol and in 10% neutral‐buffered formalin (NBF).

### 2.3 Scheduled sample collection

At approximately 4‐week intervals from the onset of the study, fish were opportunistically netted from the tanks (*n* = 10) and the netpen (*n* = 20), killed in 400 mg/L tricaine methane sulphonate (TMS, Syndel), and the fork length and weight recorded. Kidney samples were aseptically collected and preserved in 95% ethanol.

### 2.4 Histology

NBF‐fixed liver samples were dehydrated through isopropanol, clarified in xylene, blocked in paraffin and processed routinely for histological examination. Each tissue section was stained with haematoxylin and eosin or with Giemsa stain, dehydrated and sealed under a glass cover slip.

### 2.5 DNA extraction and quantitative polymerase chain reaction

Necropsy observations from moribund or dead fish suggested the presence of infection with an intracellular bacterium. Thus, DNA was extracted following the manufacturer's instructions (Qiagen DNeasy^®^ Blood and Tissue) from ethanol‐preserved liver in diagnostic cases, kidney in scheduled samples, and from cultured bacteria to be used as template in a *P. salmonis* qPCR. Once eluted, the DNA was stored at −20°C. Of the scheduled samples, on each date all 10 from the RSW and UVSW groups and 10 randomly selected from the NP group were subjected to qPCR. To assess a possible role of wild pink salmon as a reservoir of *P. salmonis* infection, archival DNA samples, extracted from kidneys of 100 pink salmon and stored at −20°C, were screened in the qPCR assay. The pink salmon had been collected between 2011 and 2015 (*n* = 20 per year) from wild spawning populations in the Quinsam River, BC, approximately 150 km north of PBS on Vancouver Island. These populations included naturally spawned and enhanced salmon, the latter being released from the hatchery as fry immediately following swim‐up.

The qPCR assay used published primer and probe sequences to amplify a sequence of bacterial 23S rDNA (Corbeil, McColl, & Crane, 2003). An individual reaction consisted of 1× TaqManTM Universal PCR Master Mix (Applied Biosystems), 900 nM each of the forward and reverse primer, 250 nM of the probe, 2 µl DNA template and nuclease‐free water for a final reaction volume of 25 µl. Reactions were run in duplicate on a StepOnePlus real‐time detection system (Applied Biosystems) following the manufacturer's protocol. For each qPCR run, the number of copies per reaction (c/rxn) was determined from a standard curve generated by amplifying a 10‐fold serially diluted double‐stranded DNA gBLOCK fragment (IDT Technologies) which included the *P. salmonis* primer and probe binding sites and ranged from 10^7^ to 10^1^ c/rxn. The number of genome equivalents per reaction (Geq/rxn) was estimated by dividing c/rxn by six, reflecting the sixfold replication of the 5S‐16S‐23S rRNA operon in the *P. salmonis* genome (Nourdin‐Galindo et al., 2017).

The limit of detection and quantification for the qPCR assay were determined from a series of 10‐fold dilutions of the gBLOCK from 10^5^ to 10^2^ c/rxn and then fivefold dilutions to 1 c/rxn. The mean c/rxn, standard deviation and coefficient of variation (CV) were calculated from 12 replicate qPCR reactions for each dilution. The limit of detection was defined as the dilution at which gBLOCK DNA was detected in >50% of the wells, and estimated here to be 5 c/rxn, or approximately 1 Geq/rxn. The limit of quantification (LOQ) was defined as CV ≤ 25%, and estimated here to be 50 c/rxn, or approximately 8 Geq/rxn. Only values exceeding the LOQ are reported.

### 2.6 Statistical analysis

Differences in median seawater temperature, salinity and DO levels were tested using Mann–Whitney rank‐sum tests and were considered statistically significant when *p* < .05. Kaplan–Meier survival curves and log‐rank analysis of differences in mortality were generated using the survminer package in R (Kassambara & Kosinski, 2018) which generated adjusted *p*‐values (Bonferroni) of the pairwise comparisons.

## 3 RESULTS

### 3.1 Environmental conditions

**Figure 1:**
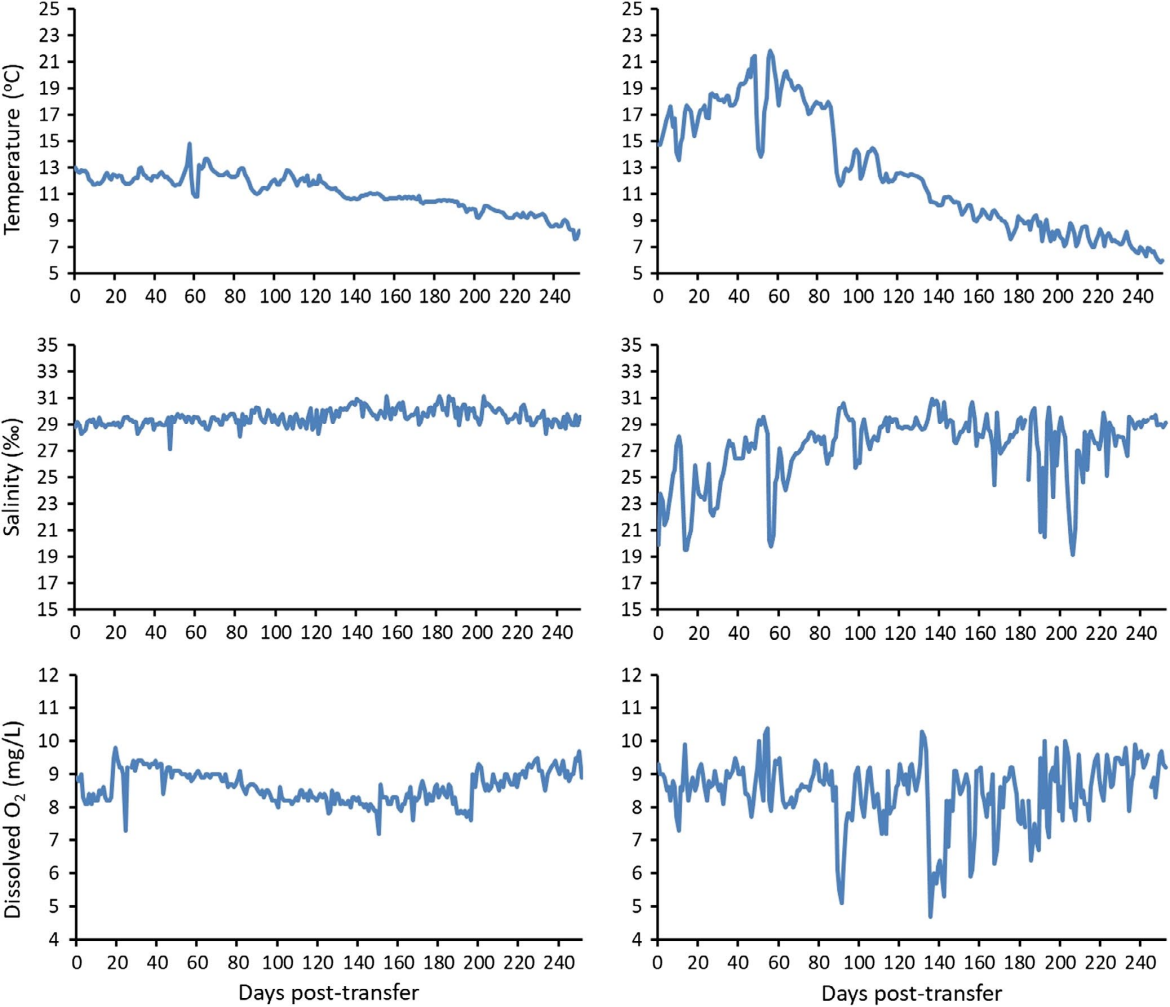
Seawater temperature, salinity and dissolved oxygen in experimental tanks at the Pacific Biological Station (left column) and in the netpen in Departure Bay (1 m depth, right column) during the course of this study [Colour figure can be viewed at http://www.wileyonlinelibrary.com]

Fish were transferred to the netpen (NP) and allocated to experimental tanks in the late spring and terminated 252 day later (13 June 2018–20 February 2019). During this time, seawater temperature, salinity and dissolved oxygen (DO) were considerably more variable in the NP at 1 m depth than in the tanks (Figure 1). Between 0 and 168 day post‐transfer (dpt), roughly when the elevated daily mortality ended, differences in conditions between the NP and tanks were statistically significant for temperature: 14.4 (9.0–21.9)°C versus 12.0 (10.6–14.8)°C (*p* < .001) and salinity: 27.9 (19.5–30.9) ‰ versus 29.4 (27.1–31.1) ‰ (*p* < .001), but not for DO: 8.6 (4.7–10.4) mg/L versus 8.6 (7.2–9.8) mg/L. A bloom of *Chaetoceros* sp. occurred between 100 and 130 dpt, with counts up to 2,800 cells/L. DO levels in the NP were suboptimal between 89 and 92 dpt (5.1–6.2 mg/L) and again at 141 and 142 dpt (5.3–5.8 mg/L) (Figure 1).

### 3.2 Mortality

**Figure 2:**
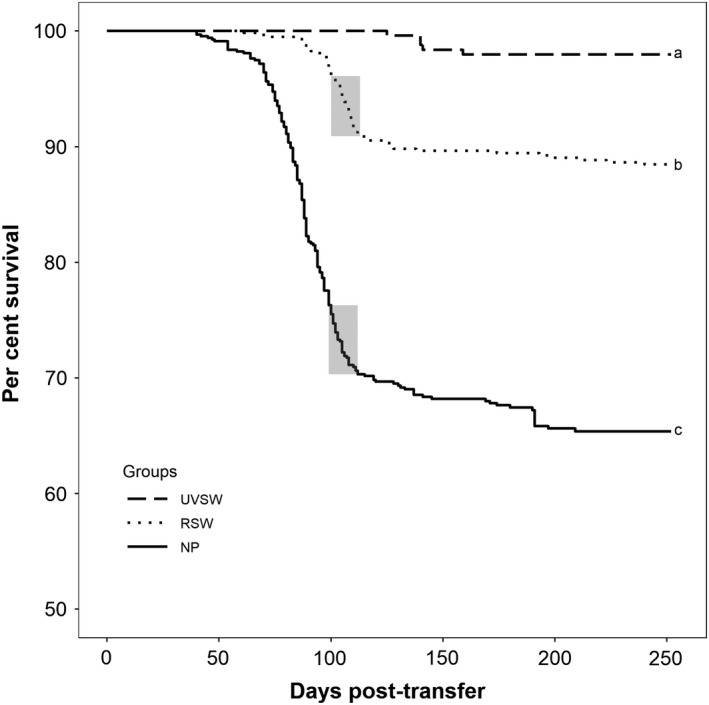
Survival of Atlantic salmon *Salmo salar* during an outbreak of piscirickettsiosis in Departure Bay, Nanaimo. Salmon were reared in an open netpen (NP) in the Bay or in tanks supplied with raw (RSW)‐ or UV‐irradiated sea water (UVSW) supplied from the Bay. Shaded areas indicate oral treatment with oxytetracycline (100 mg kg^−1^ day^−1^). Letters after each survival curve reflect statistical significance (*p* < .05)

The first mortalities in the NP occurred at 39 dpt, roughly 2 weeks after the average daily temperature reached 20°C, and cumulative mortality was 33.9%. The first mortality in the raw seawater (RSW) tanks occurred at 61 dpt, and cumulative mortality in this treatment was 11.5% (Figure 2). Over the course of the eight‐month trial, five fish in the UVSW tanks died (1.7%). Survival in the NP population was significantly lower compared with survival in either RSW (*p* < .001) or UVSW (*p* < .001). Similarly, survival in the RSW tanks was significantly lower compared with that in the UVSW tanks (*p* < .001).

### 3.3 Necropsy

**Figure 3:**
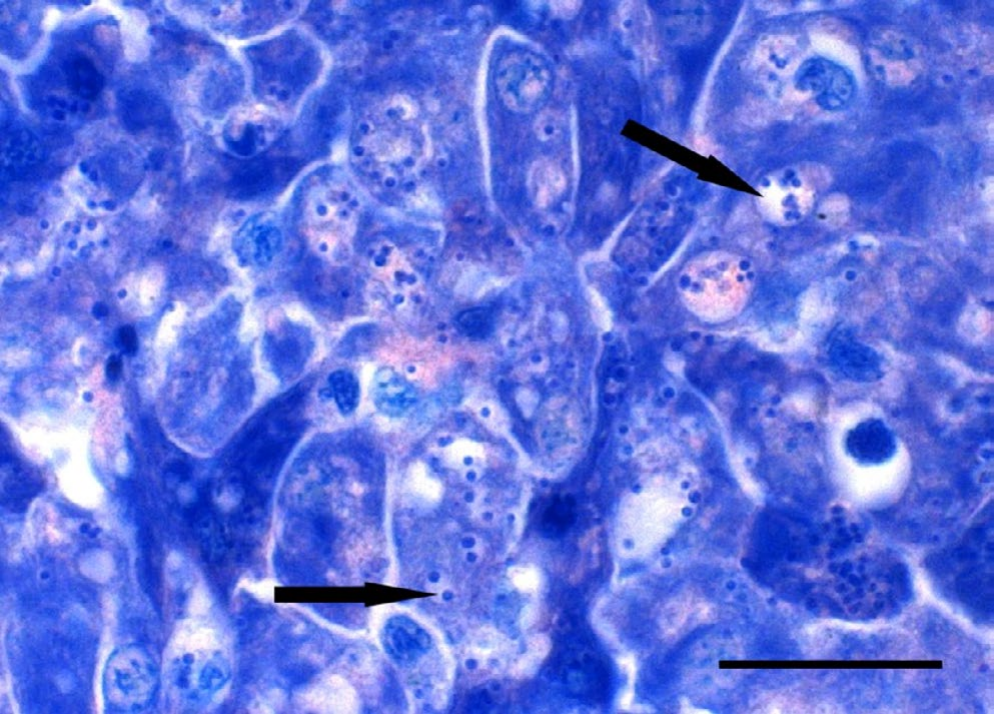
Histological section of liver from Atlantic salmon (*Salmo salar*) infected with *Piscirickettsia salmonis*. Vacuolated hepatocytes, showing numerous bacterial cells, often within pale‐staining vacuoles (arrows). Giemsa stain. Scale bar is 20 µm [Colour figure can be viewed at http://www.wileyonlinelibrary.com]

Carcasses submitted from the NP at 78 dpt (*n* = 4) gave inconclusive results because of an absence of gross lesions exacerbated by autolysis. Of the 10 submitted at 88 dpt, principal findings were flank skin ulceration (*n* = 6), buccal skin ulceration (*n* = 5), splenomegaly (*n* = 5), gastric distension with fluid (*n* = 3), swollen kidney (*n* = 3), hyphema (*n* = 3) and pale circular subcapsular zones of discolouration of liver with focal congestion (*n* = 2). Gram‐stained impression smears were positive for diplococci in liver (*n* = 6), kidney (*n* = 3) and skin ulcers (*n* = 3 of 3). Autolysis hindered the interpretation of most histological sections; however, Giemsa‐stained sections of well‐fixed liver revealed foci of hepatic necrosis and degeneration, with intracellular bacteria (Figure 3). None of 10 kidney impressions from moribund or fresh‐dead fish were positive for *R. salmoninarum* by DFAT. All 10 liver samples from moribund or fresh‐dead fish tested positive for *P. salmonis* by qPCR (range: 0.004–4,710.1 copies/ng DNA). Growth was observed on BCG agar streaked with kidney or liver. Pinpoint colonies first observed after 4 day were pale grey and after 12 day were 1–2 mm in diameter with translucent, smooth borders. DNA extracted from the colonies tested positive for *P. salmonis* by qPCR.

Carcasses submitted from the RSW tanks at 99 dpt (*n* = 3) presented with gross, white hepatic nodules, diplococci in Gram‐stained impressions of kidney and liver. Growth on BCG agar from kidney and liver tested positive for *P. salmonis* by qPCR (range: 43.9–7,816.8 copies/ng DNA). One of five fish that died in the UVSW tanks was tested and the carcass found to be negative for *P. salmonis* by qPCR.

### 3.4 Treatment

A 14‐day course of oral oxytetracycline (100 mg/kg fish/day) was initiated following qPCR confirmation of *P. salmonis* in the NP (at 98 dpt) and the RSW tank (at 99 dpt) populations, with corresponding declines in cumulative mortality (Figure 2).

### 3.5 Scheduled sampling

*Piscirickettsia salmonis* was most frequently detected by qPCR in kidney samples collected 84 dpt. At this time, positive qPCR results were obtained from eight of 10 RSW fish and from one of 10 NP fish (Table 1). At all other sample times between 28 and 252 dpt, positive results were obtained from four of 70 NP samples and from one of 60 RSW samples. No samples from the UVSW tanks tested positive for the bacterium at any time.

**Table 1:** The prevalence and relative abundance (mean copies/ng DNA of samples exceeding the LOQ) of *Piscirickettsia salmonis* 16S rDNA in kidney of Atlantic salmon (*Salmo salar*) following transfer to an open netpen (NP) or to tanks supplied with raw sea water (RSW) or UV‐irradiated sea water (UVSW) at the Pacific Biological Station, Nanaimo, British Columbia

Days post‐transfer	Location	#Pos/#Test	Copies/ng DNA
0	Pretransfer	0/10	
28	NP	0/10	
	RSW	0/10	
	UVSW	0/10	
56	NP	2/10	0.980
	RSW	1/10	0.016
	UVSW	0/10	
84	NP	1/10	0.336
	RSW	8/10	0.515
	UVSW	0/10	
112	NP	1/10	0.080
	RSW	0/10	
	UVSW	0/10	
140	NP	0/10	
	RSW	0/10	
	UVSW	0/10	
168	NP	1/10	0.665
	RSW	0/10	
	UVSW	0/10	
252	NP	0/20	
	RSW	0/10	
	UVSW	0/10	

Of 100 kidney samples screened from the Quinsam River pink salmon, *P. salmonis* was detected in one of 20 collected in 2013 (11.25 copies/ng DNA).

## 4 DISCUSSION

An outbreak of piscirickettsiosis (SRS) occurred in Atlantic salmon smolts maintained in Departure Bay sea water during the summer and autumn of 2018. In addition to elevated mortality, affected fish displayed gross and microscopic lesions typical of SRS, and bacteria isolated from these fish were confirmed to be *P. salmonis* by qPCR. Additionally, *P. salmonis* DNA was detected in liver samples collected from dead and moribund fish and in kidney from scheduled samples by qPCR. Mortality in NP‐ and tank‐reared fish was slowed by oxytetracycline, consistent with the successful application of this antibiotic to treat previous outbreaks of SRS in BC farmed salmon (Brocklebank et al., 1993, 1992). The data indicate that transmission of the bacterium to the NP‐reared fish occurred directly from the surrounding sea water. Similarly, infection among the tank‐reared salmon indicated transmission via incoming raw sea water (RSW) despite sand filtration.

The delayed onset of the SRS outbreak in the tank‐reared salmon may be accounted for by the more stable environmental conditions involving reduced temperature and elevated salinity, in comparison with the NP‐reared salmon. It is unlikely that this delayed onset was explained by a reduction in the seawater concentration of *P. salmonis* due to sand filtration, given that the filter in use at PBS removes particles greater than 68 µm (Jones, Cho, Nguyen, & Mahony, 2016). The relatively stable water quality parameters of the sea water in the tanks reflected the deep intake from Departure Bay. These conditions may have also contributed to the elevated survival in the tank‐reared salmon; however, these fish also benefited from treatment at an earlier stage of the disease outbreak due to the earlier diagnosis in the NP‐reared salmon. The earlier onset and increased SRS‐associated mortality in the NP‐reared salmon held under more variable conditions were similar to that in aquaculture in which outbreaks of the disease have been associated with elevated water temperatures (Rees et al., 2014), algal blooms and severe meteorological events (Cusack, Groman, & Jones, 2002; Olsen, Melby, Speilberg, Evensen, & Hastein, 1997). Infection with multiple pathogens may increase the severity of disease in fish (Long, Garver, & Jones, 2019); however, there was no evidence of co‐infection or pathology of sufficient intensity or severity to explain the increased mortality in the NP‐reared salmon. Rather, our findings support a *P. salmonis* aetiology for the elevated mortality in tank‐ and NP‐reared salmon that in the latter, was exacerbated by variable environmental conditions.

In contrast to our ability to detect *P. salmonis* in morbid or dead fish, the infection was detected in a relatively small number of scheduled samples, emphasizing our uncertainty about the incidence of infection during the course of the disease. This uncertainty was best illustrated in samples collected at 84 dpt, in which the bacterium was detected by qPCR in eight of 10 RSW fish, in which the cumulative mortality to date was 1%, and in only one of 10 NP fish, in which the cumulative mortality to date was 12%. In contrast, the absence of infection in most of the subsequent samples appeared to reflect treatment efficacy. Survival in the RSW tank‐reared populations was significantly greater than in the NP despite biomass densities in the tanks exceeding that in the netpen by almost a factor of 10, suggesting that in this study, environmental instability played a greater role than fish density in triggering SRS and its subsequent severity. Further controlled studies are required to test this hypothesis.

The absence of *P. salmonis* infection and SRS in UVSW‐reared salmon suggested that the bacterium present in RSW was inactivated by the UV treatment. At the PBS aquarium, RSW is irradiated with UV at a wavelength of 265 nm and doses ranging from 317 to 721 mJ/cm^2^, well above doses known to inactivate a range of fish pathogenic bacteria (Bullock & Stuckey, 1977; Liltved, Hektoen, & Efraimsen, 1995). Inactivation studies of *P. salmonis* have focused on the application of disinfectants (Muniesa et al., 2018), and there are no published accounts of UV inactivation. Although more work is required to better establish dose and time parameters related to UV inactivation, the present study indicates the potential for treatment of sea water with UV as an aid in *P. salmonis* biosecurity. Elsewhere, we have demonstrated that the UV‐irradiation system described here inactivates the infectivity of *Kudoa thyrsites*, a myxozoan parasite of salmon which is enzootic in Departure Bay sea water (Jones et al., 2016; Jones & Long, 2019).

The data strongly indicate a natural reservoir of the *P. salmonis* infection in Departure Bay sea water; however, the identity of this source has not been established. Between 1970 and 2005, prior to the installation of the UV‐sterilization system at PBS, 9 cases of *P. salmonis*‐like infections were diagnosed in pink, Chinook or chum salmon (*Oncorhynchus keta*) reared on sea water in the research aquarium (DFO‐PBS Diagnostic Laboratory, unpublished data). All but one of these cases occurred between August and February, and in no case was a source identified. Pink salmon are enhanced locally to support a sport fishery, and over the last 14 years, an annual average of 266,782 pink salmon fry (range: 101,520–400,255) have been reared in an open NP adjacent to the site of the present experimental NP. Every spring, fish are held for 4–5 weeks and then released to rear in the ocean whence they return a year later as adults. Beginning mid‐August 2018, adult pink salmon were angled in Departure Bay, approximately 1 km from the NP and aquarium seawater intake. Although the number of pink salmon and their infection status was not determined, the timing and proximity of this migration in 2018 suggested the pink salmon as a possible source of the infection. The detection of *P. salmonis* in one of 100 spawning pink salmon from the Quinsam River, while confirming the infection naturally occurs in this species, did little to support the hypothesis that the pink salmon enhancement activities contribute to an increased risk of *P. salmonis* in Departure Bay. Monitoring for the infection in pink salmon returning to Departure Bay is warranted. In Chile, the bacterium was detected in 44% of 16 apparently healthy coho salmon during the upstream spawning migration (Pérez, Alert, Contreras, & Smith, 1998).

An outbreak of SRS is reported in naïve Atlantic salmon within a few weeks of transfer to sea water and associated with elevated mortality, morbidity and characteristic lesions. The infection and disease were absent in salmon held in UV‐irradiated sea water, and the severity of SRS was lower among fish held in deep‐sourced water that was cooler, more saline and more consistently well‐oxygenated compared with fish in a netpen near the surface. The absence of adequate vaccine protection against this disease combined with a poor understanding of the reservoirs of infection emphasizes the risk of this marine‐sourced infectious disease faced by open netpen salmon aquaculture.

## CONFLICT OF INTEREST

The authors declare no conflict of interest.

## Data Availability

The data that support the findings of this study are available from the corresponding author upon reasonable request.
